# High-Definition Transcranial Direct Current Stimulation-Primed Intermittent Theta Burst Stimulation in Treatment of Negative Symptoms of Schizophrenia - A Randomised Sham Controlled Study

**DOI:** 10.1192/j.eurpsy.2025.296

**Published:** 2025-08-26

**Authors:** R. Gupta, A. Sharma

**Affiliations:** 1 Psychiatry, Central Institute of Psychiatry, Ranchi, Ranchi, India

## Abstract

**Introduction:**

Negative symptoms of Schizophrenia (NSS) consists of apathy, avolition, anhedonia, social withdrawal, reduced expression (blunted affect and alogia) and impairments in attention, working memory, or executive function. Unfortunately, treatment effects of conventional approaches with antipsychotics, other pharmacological agents or psychosocial interventions are limited and have not shown much improvement in reducing negative symptoms. Non-Invasive brain stimulation such as repetitive transcranial magnetic stimulation (rTMS) & high-definition transcranial direct current stimulation (HD-tDCS) offers a novel approach in the treatment of negative symptoms but with no agreed upon treatment protocol. Priming is a technique that can enhance the sensitivity of the brain to therapy using techniques that increase or decrease the excitability of the cortex. Preconditioning TDCS have shown profound impact on the conditioning effects induced by subsequent administration of rTMS in a few studies done in patients with depression. There has been no study assessing the effects of HD-tDCS primed iTBS in Negative Symptoms of Schizophrenia.

**Objectives:**

The aim of this study was to evaluate whether HD-tDCS priming improves the efficacy of intermittent theta burst stimulation (iTBS) in improving the NSS.

**Methods:**

A prospective hospital based randomized controlled study with a sample size of 40, where the participants were divided into Active or Sham HD-tDCS primed iTBS stimulation groups for a total of 20 sessions over two weeks. Assessments on clinical parameters at baseline, end of 2 weeks, and end of 4 weeks were done.

Primary outcome of the study was the differences in Positive and Negative Syndrome Scale for Schizophrenia (PANSS) & Scale for Assessment of Negative Symptoms (SANS) scores over 4 weeks of HD-tDCS primed iTBS between the two groups.

**Results:**

There was a statistically significant reduction noted in the mean PANSS & SANS scores across all domains over time up to 4 weeks in both the active and sham groups.

Though there was more reduction in mean scores in total PANSS score as well as across domains, the difference between the two groups was not found to be statistically significant in any of the PANSS score. However, the difference was found to be significant for SANS total score and SANS attention score with score showing more reduction in the active group in comparison to the sham group.

**Conclusions:**

These results suggest that the HD-tDCS primed iTBS had a superior effect specifically on negative symptoms, as reflected in the SANS scores, but not on the broader spectrum of symptoms measured by PANSS, which suggests that priming iTBS with HD-tDCS may prove to be beneficial in reducing the Negative Symptoms of Schizophrenia more effectively than iTBS alone.

**Disclosure of Interest:**

None Declared

